# Comprehensive analysis of prognostic immune-related genes and drug sensitivity in cervical cancer

**DOI:** 10.1186/s12935-021-02333-9

**Published:** 2021-12-01

**Authors:** Ya-Nan Pi, Jun-Nan Guo, Ge Lou, Bin-Bin Cui

**Affiliations:** 1 Department of Gynecology, Harbin Medical University Cancer Hospital, Harbin, 150086 P. R. China; 2 Department of Colorectal Surgery, Harbin Medical University Cancer Hospital, Harbin, 150086 P. R. China

**Keywords:** Cervical cancer (CC), Immune-related genes (IRGs), Prognosis, Prognostic risk score model (PRSM), Drug sensitivity

## Abstract

**Background:**

Cervical cancer (CC) is the leading cause of cancer-related death in women. A limited number of studies have investigated whether immune-prognostic features can be used to predict the prognosis of CC. This study aimed to develop an improved prognostic risk scoring model (PRSM) for CC based on immune-related genes (IRGs) to predict survival and determine the key prognostic IRGs.

**Methods:**

We downloaded the gene expression profiles and clinical data of CC patients from the TCGA and GEO databases. The ESTIMATE algorithm was used to calculate the score for both immune and stromal cells. Differentially expressed genes (DEGs) in different subpopulations were analyzed by “Limma”. A weighted gene co-expression network analysis (WGCNA) was used to establish a DEG co-expression module related to the immune score. Immune-related gene pairs (IRGPs) were constructed, and univariate- and Lasso-Cox regression analyses were used to analyze prognosis and establish a PRSM. A log-rank test was used to verify the accuracy and consistency of the scoring model. Identification of the predicted key IRG was ensured by the application of functional enrichment, DisNor, protein–protein interactions (PPIs) and heatmap. Finally, we extracted the key prognostic immune-related genes from the gene expression data, validated the key genes by immunohistochemistry and analyzed the correlation between their expression and drug sensitivity.

**Results:**

A new PRSM was developed based on 22 IRGPs. The prognosis of the low-risk group in the model group (*P* < 0.001) and validation group (*P* = 0.039) was significantly better than that in the high-risk group. Furthermore, M1 and M2 macrophages were highly expressed in the low-risk group. Retinoic acid-inducible gene-I (RIG-I)-like receptors (RLRs) and the Janus kinase-signal transducer and activator of transcription (JAK-STAT) signaling pathway were significantly enriched in the low-risk group. Three representative genes (CD80, CD28, and LCP2) were markers of CC prognosis. CD80 and CD28 may more prominent represent important indicators to improve patient prognosis. These key genes was positively correlated with drug sensitivity. Finally, we found that differences in the sensitivity to JNK inhibitors could be distinguished based on the use and risk grouping of this PRSM.

**Conclusions:**

The prognostic model based on the IRGs and key genes have potential clinical significance for predicting the prognosis of CC patients, providing a foundation for clinical prognosis judgment and individualized treatment.

**Supplementary Information:**

The online version contains supplementary material available at 10.1186/s12935-021-02333-9.

## Introduction

Cervical cancer (CC) is the second most frequently diagnosed cancer and the fourth cause of cancer mortality in women [1]. In 2020, there were an estimated 604,000 new cases, and 342,000 deaths due to CC worldwide [1]. While most patients with early-stage CC can be cured with surgery, the main treatment for locally-advanced CC is concurrent radiotherapy and chemotherapy. However, no significant improvement has been observed for the treatment of persistent or recurrent CC, which cannot be controlled by surgery or chemo-radiotherapy. The five-year overall survival (OS) of patients with III and IV stage CC was 52.0% and 29.8%, respectively, and the relapsed patients was only 15% [2]. At the same time, the clinical outcomes of the same-stage patients differed due to tumor heterogeneity [3]. Thus, the identification of prognostic biomarkers for CC has great significance for improving the survival rate of patients and promoting the development of precision therapy.

A large number of studies investigating cancer have shown that the main reason for tumor heterogeneity is the complex cellular composition of the tumor microenvironment (TME). The composition and level of immune-related cells plays a key role in the development and prognosis of cancer, as well as the response to immunotherapy [4]. Over the past decade, surgery, chemotherapy, and/or radiotherapy have remained the primary treatment for CC. However, immunotherapy has become a novel strategy and is associated with many key advances [5]. Further evidence has preliminarily confirmed that immune-prognostic features can be used to predict the prognosis of CC [6, 7]. However, it remains necessary to further identify more accurate prognosis-related immune genes that can predict patient prognosis and increase the development of novel immunotherapy for CC.

In this study, we aimed to investigate the correlation between immune-related characteristics and genes associated with CC prognosis. We constructed immune-related gene pairs (IRGPs) by analyzing gene expression profiles in the TCGA database, which was used to establish and verify the prognostic risk scoring model (PRSM), and calculated the risk score (RS). Next, the patients were divided into high and low risk groups to study the biological functions and signaling pathways associated with the differentially enriched genes. Therapeutic sensitivity was also analyzed, which included chemotherapy agents and targeted inhibitors (TIs). Finally, we identified key immune-related genes (IRGs) for CC prognosis, and evaluated the value of the predictive model and the key prognostic immune-related genes for the clinical treatment of CC.

## Materials and methods

### Data collection

In this study, transcriptome data from the TCGA (https://portal.gdc.cancer.gov/) and GEO (http://www.ncbi.nlm.nih.gov/geo/) high-throughput platforms were acquired 257 and 300 CC samples from TCGA and GEO, respectively. After screening, 515 CC samples with complete clinical information were included in the analysis, which was comprised of 253 TCGA and 262 GEO samples. For each dataset, the Gene ID was converted to the corresponding gene symbol according to its corresponding annotation package. The TCGA data was termed the model group, and the GEO data was termed the validation group. The analysis excluded RNA which was not detected in 10% of the samples.

### Identification of differentially expressed IRGs

R package “ESTIMATE” was used to assess the extent of immune infiltration in the samples. Based on the SSGSEA algorithm, the tumor expression matrix of each sample was scored using stromal and immune gene sets [8]. We then used R package “maxstat” to calculate the optimal cut-off value of the immune and stromal scores, and divided all of the samples into high and low rating groups [9]. Using the Kaplan–Meier method for visualization, the difference between the survival curves in the high and low rating groups was calculated using the logarithmic rank test of R package “survcomp” [10]. The R package “Limma” [11] was used to analyze differentially expressed genes in different subsets of samples (DEGs) (|log2foldchange|> 0.5, *P*-adj < 0.05). Subsequently, the intersection of the two groups of up-regulated and down-regulated DEGs were obtained to screen out the IRGs in CC and display them in a Venn diagram.

### Identification of gene consensus modules

We used a weighted gene co-expression network analysis (WGCNA) [12] to construct consensus gene modules for DEGs, and analyzed the correlation between the module and the “ESTIMATE” results. First, we used powers to build an adjacency matrix (AM). We selected the appropriate power index to increase the matrix similarity to construct a scale-free co-expression network. The AM was then converted into a topological overlap matrix (TOM). Based on the TOM dissimilarity measurements, we conducted an average linkage hierarchical cluster analysis. Among them, a correlation coefficient was defined as the correlation between the characteristic genes of each module and the associated scores. Gene significance (GS) was defined as the *P*-value in the linear regression of the expression and score for each gene (GS = lgP). Finally, we obtained the gene cluster tree, co-expression module, and corresponding correlation.

### Establishment of immune-related gene pairs (IRGPs)

To further screen immune-related genes in order to establish immune gene pairs, we selected the module with the highest correlation coefficient, and the GS and module membership (MM) were calculated. MM is an indicator that measures gene and module connectivity. We defined the threshold for filtering as: cor. gene MM > 0.6 and cor. gene GS > 0.6. To eliminate the sequencing errors between the different platforms and samples, we constructed gene pairs from immune-related genes that were screened out. Specifically, the expression of two genes was compared in each sample; if the expression of the former is greater than the latter, it was denoted as 1, otherwise, it was 0. After removing the IRGPs with minimal expression and an uneven distribution (MAD = 0), a univariate Cox proportional hazards regression analysis was performed for the remaining IRGPs in the model group. Statistically significant IRGPs from the univariate Cox analysis were retained for a subsequent Lasso-Cox proportional hazards regression analysis with 1000 simulations using R package “glmnet” [13]. R package “survivalROC” [14] was used to draw 3-, 5-, and 10-year time-dependent receiver operating characteristics (timeROC) and determine the area under the curve (AUC). The optimal cut-off value was selected on the ROC curve with the maximum AUC. Finally, the predictive model was applied to the validation group and all patients were divided into low-risk groups using cut-off values.

### Validation of the predictive model

A log-rank test was used to analyze the prognosis of patients in the model group and the validation group at high and low risk, to verify the accuracy and consistency of the scoring model. In the model group with complete clinical information, both the univariate and multivariate Cox regression analyses were performed with a risk score combined with other clinical factors, and the independent effect of the risk score was further verified.

### Exploration of the differences in immune infiltration

Another algorithm was used to estimate the relative infiltration abundance of 22 immune cells in different samples with R package “CIBERSORT” [15], and differences in immune infiltration between the high- and low-risk groups were analyzed. The samples with *P* < 0.05 in the calculation results were retained, and a difference analysis in the content of the various immune cells in the high- and low-risk groups was determined using a Wilcoxon rank sum test.

### Functional enrichment analysis

To investigate the differentially enriched biological functions and signaling pathways in the high- and low-risk groups, we used bioconductor package “fgsea” [16] to conduct a gene ontology (GO)- and kyoto encyclopedia of genes and genomes (KEGG)-related gene set enrichment analysis (GSEA) with 10,000 permutations. To compare the genes between the high- and low-risk groups, the ratio of gene expression was sequenced by a log_2_ multiple conversion. The threshold values were *P* < 0.05.

### Identification of key prognostic immune-related genes

For further screen for key prognostic immune-related genes, we performed a protein interaction network analysis on the module genes used to construct immune gene pairs using STRING (https://www.string-db.org). Genes with more than 10 nodes in the network were selected to create an intersection with the genes in the model, and the prognosis of the intersection genes was analyzed. After screening out key prognostic immune-related genes, we used the DisNor database (https://disnor.uniroma2.it/) to analyze and search for their upstream- and downstream-related genes, as well as the mode of action. DisNor is a disease-focused resource that uses the causal interaction information annotated in SIGNOR, and the protein interaction data in Mentha were used to generate and explore protein interaction networks that link disease genes. Heatmap was plotted using R package “pheatmap”.

### Immunohistochemistry

Immunohistochemistry (IHC) was used to verify the expression of CD80 and CD28 in CC and adjacent normal tissues. Tissue microarrays were purchased from the Wuhan servicebio technology CO., LTD, which are predominantly intended for commercialization. The tissue microarrays include 11 samples of cervical cancer tissue and 11 samples of adjacent normal tissues (IWLT-N-22CC81, CC-1801, servicebio) (Table 1). The pathological types of cervical cancer were squamous cell carcinoma. The tissue microarrays assay (TMA) can be used to validate bioinformatics data. Immunohistochemical staining was performed with the antibody against CD80 (1:100, 66406-1-lg, Proteintech) and antibody against CD28 (1:100, 65099-1-lg, Proteintech). Tissue sections were dewaxed to water, and placed in a repair box filled with EDTA (PH9.0) antigen repair solution (G1203, servicebio) for antigen repair. Slides were incubated with primary antibody at 4 °C overnight, followed incubation with secondary antibody for 50 min at 37 °C, counter-stained with 10% Mayer’s hematoxylin, dehydrated, and mounted. IHC staining was assessed by summing intensity and quantity scores. Intensity score was graded as 0 (negative), 1 (weakly positive, light brown), 2 (moderately positive, brown), or 3 (strongly positive, dark brown). Quantity score was graded as 0 (negative), 1 (≤ 25%), 2 (26–50%), 3 (51–75%), or 4 (> 75%). IHC results were evaluated by two experienced pathologists independently. In cases of disagreement, a third pathologist made the judgment.

**Table 1:** Clinicopathological date on patients of tissue microarrays

No.	Chip No.	Category	Histological type	Differentiation degree	TNM (clinical, 9th edition)	Stage (FIGO 2018)
1	A1, 2	1: Carcinoma, 2: Adjacent normal tissues	Squamous cell carcinoma	Medium—low	T1b3N1M0	IIIC1
2	A3, 4	3: Carcinoma, 4: Adjacent normal tissues	Squamous cell carcinoma	Medium	T1b3N0M0	IB3
3	A5, 6	5: Carcinoma, 6: Adjacent normal tissues	Squamous cell carcinoma	Medium	T1b3N0M0	IB3
4	B1, 2	1: Carcinoma, 2: Adjacent normal tissues	Squamous cell carcinoma	Medium	T1b3N0M0	IB3
5	B3, 4	3: Carcinoma, 4: Adjacent normal tissues	Squamous cell carcinoma	Low	T1b2N0M0	IB2
6	B5, 6	5: Carcinoma, 6: Adjacent normal tissues	Squamous cell carcinoma	Medium–low	T1b2N1M0	IIIC1
7	C1, 2	1: Carcinoma, 2: Adjacent normal tissues	Squamous cell carcinoma	Low	T1b3N1M0	IIIC1
8	C3, 4	3: Carcinoma, 4: Adjacent normal tissues	Squamous cell carcinoma	Low	T1b2N0M0	IB2
9	C5, 6	5: Carcinoma, 6: Adjacent normal tissues	Squamous cell carcinoma	Medium–high	T1b2N0M0	IB2
10	D1, 2	1: Carcinoma, 2: Adjacent normal tissues	Squamous cell carcinoma	Low	T1b3N0M0	IB3
11	D3, 4	3: Carcinoma, 4: Adjacent normal tissues	Squamous cell carcinoma	Medium	T1b3N1M0	IIIC1

### Sensitivity analysis of different treatments

To evaluate the value of the predictive models and key prognostic immune-related genes in the clinical management of CC, we analyzed the treatment sensitivity from two aspects: chemotherapy drugs and TIs. We used R package “pRRophetic” [17] to calculate the concentration of 50% reduction growth (IC50) caused by TIs, including vascular endothelial growth factor receptor (VEGFR), Hedgehog (HH), and Wnt inhibitors. A Wilcoxon rank sum test was used to compare the IC50 differences between the various risk groups.

In addition, we downloaded the gene expression and chemotherapeutic drug response data from CellMiner™ (https://discover.nci.nih.gov/cellminer/). These data were also from the same batch. We removed the drugs that were not approved by the (Food and Drug Administration) FDA or in clinical trials. We then extracted the key prognostic immune-related genes from the gene expression data, and analyzed the correlation between their expression and drug sensitivity.

### Statistical analyses

All statistical analyses were managed by R software (Version 3.6.3) and SPSS (Version 25). The unpaired t-test was used to compare the means of two groups, and a two-tailed p value of < 0.05 was considered statistically significant.

## Results

### Identification of DEGs based on the immune score and stromal score

**Fig. 1:**
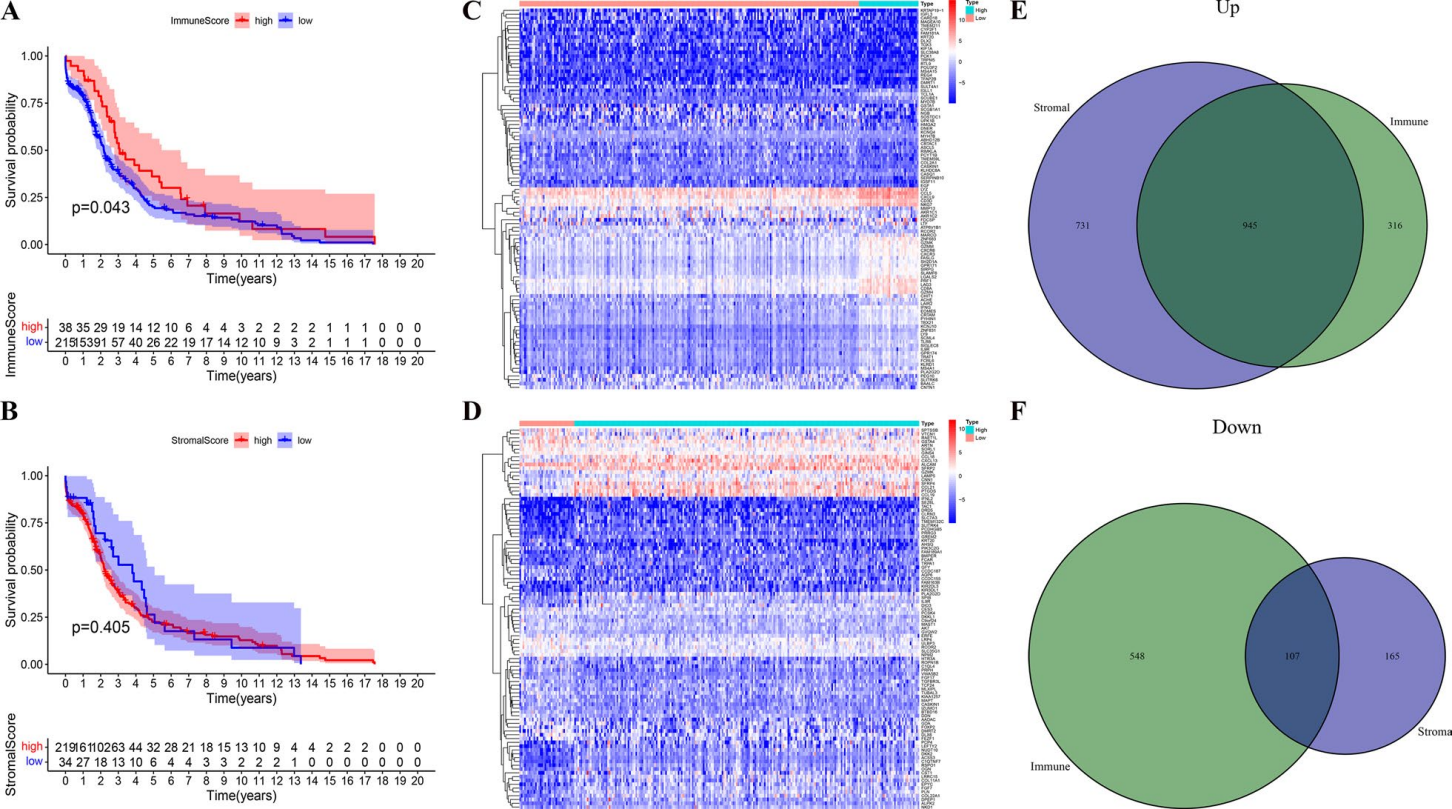
Comparison of gene expression profile with immune and stromal scores of cervical cancer. **A** Survival curve for the high and low immune score groups of CC patients. **B** Survival curve for the high and low stromal score groups of CC patients. **C** Heatmap of differentially expressed genes from the high and low immune score groups. **D** Heatmap of differentially expressed genes from the high and low stromal score groups. **E**, **F** Venn diagram analysis of aberrantly expressed genes based on immune and stromal scores

After obtaining the expression profile data of 253 CC patients from the TCGA database, the ESTIMATE algorithm was used to calculate the immune score and stromal score (Additional file 1: Table S1) to execute a prognostic analysis. The results showed that there was a significant difference in the prognosis between the groups with high and low immune scores and stromal score (*P* = 0.043) (Fig. 1A and B). To screen out the immune-related genes, we analyzed the differences in the genes from the high and low immune and stromal score groups and the results were presented in a heatmap (Fig. 1C and D). The intersection of the two groups of up-regulated and down-regulated differential genes was selected respectively (Fig. 1E and F). In the end, 1052 intersecting DEGs were screened, including 945 up-regulated and 107 down-regulated DEGs.

### Screening the most significant immune-related genes by WGCNA

**Fig. 2:**
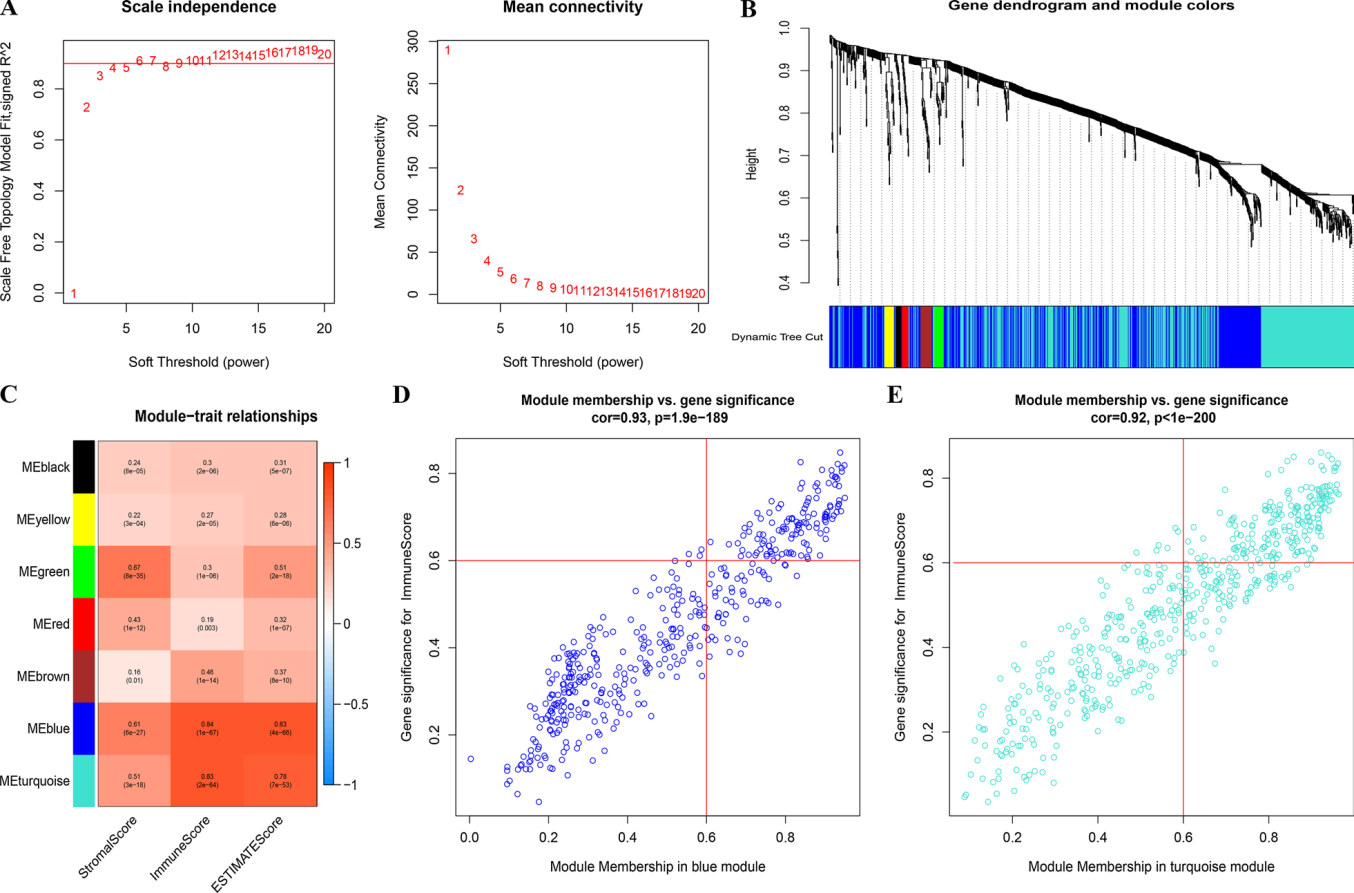
Weighted cervical cancer gene co-expression network. **A** The scale-free fit index for soft-thresholding powers. We selected a power index = 6 as the appropriate soft threshold. **B** A dendrogram of the differentially expressed genes clustered based on different metrics. Each branch in the figure represents one gene, and every color below represents one co-expression module. **C** Correlation between the gene modules and tumor microenvironment related scores, including immune score, stromal score and estimate score. Each cell contains corresponding correlation coefficient and p-value. **D** Scatter plot of module eigengenes in the blue and turquoise modules

To further screen for the immune-related genes in CC, we used WGCNA to construct gene networks to identify important immune-related gene modules and further understand the genes that induce differences in immune infiltration in CC. We selected a power index = 6 as the appropriate soft threshold (Fig. 2A), and used the 1052 intersection DEGs obtained above to construct a scale-free co-expression network and obtained a gene cluster tree and different modules (Fig. 2B). The correlation analysis of the modules and various types of fractions were used to obtain the correlation coefficient (CC) and P values (Fig. 2C). Both the blue modules (CC = 0.84, *P* < 0.001) (Fig. 2D) and turquoise modules (CC = 0.83, *P* < 0.001) (Fig. 2E) had a high correlation with the immune score. Therefore, we selected the genes in these two modules for subsequent analysis. To further screen for the key immune-related genes, we screened the genes in these two modules in accordance with the above threshold values for MM and GS, finally obtaining 301 IRGs.

### PRSM establishment and verification

**Fig. 3:**
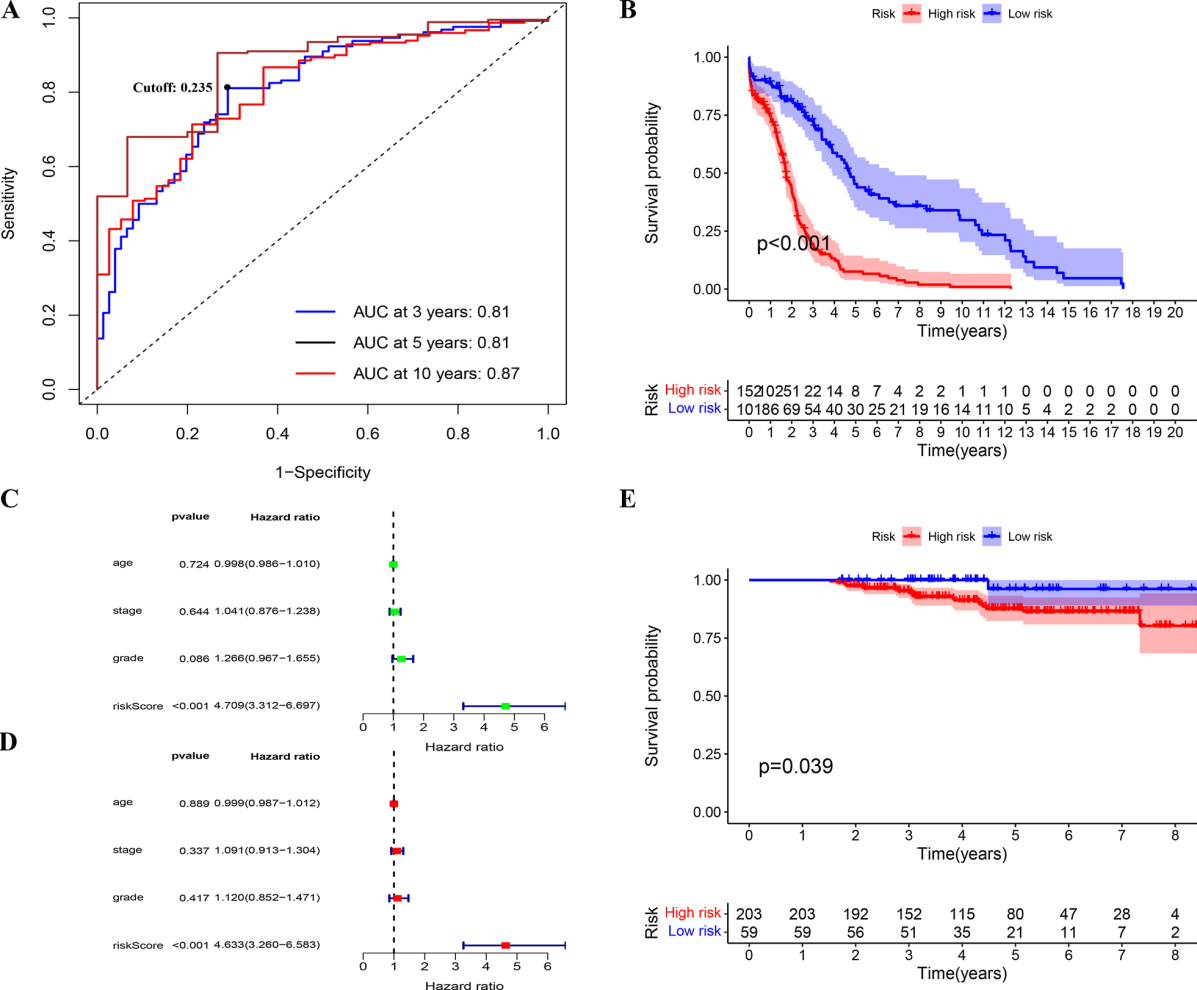
Establishment of immune-related gene pairs (IRGPs) and prognostic risk scoring model (PRSM). **A** TimeROC curves for 3, 5, 10 years were plotted in the model group. **B** KaplanMeier curve of overall survival in model group. **C** Forest plot for Univariate-Cox regression analyze in model group. **D** Forest plot for Multivariate-Cox regression analyze in model group. **E** Kaplan–Meier curve of overall survival in validation group

A total of 301 IRGs were arranged and combined, and a total of 45,150 IRGPs were established. We removed IRGPS, which had less variation (0 or 1 < 20%) in all samples, and the rest of the IRGPS were used in the univariate Cox proportional hazards regression analysis. The results showed that 35 IRGPs had significant prognostic differences (P < 0.05) (Additional file 1: Table S2). Next, we performed a lasso-cox proportional hazards regression analysis on these IRGPs, and obtained 19 prognostic IRGPs and their risk coefficients (Table 2). Using the PRSM, we obtained the risk score of all patients in TCGA and GEO databases. Moreover, we constructed a time-dependent ROC curve for the patients in the model group, and obtained the AUC values for 3, 5, and 10 years (Fig. 3A), respectively, suggesting that this prediction model exhibits good prognostic classification ability. We calculated the optimal cut-off value to be 0.235 based on the 5-year ROC curve and divided all patients into high- and low-risk groups. Survival curves were drawn for both the model group and validation group, and the results showed that the prognosis of patients in the low-risk group significantly differed (Fig. 3B and E). This finding supported the high accuracy and consistency of the PRSM. In addition, univariate and multivariate Cox analyses of risk scores combined with clinical information were performed in the model group. The results showed that the risk score had an independent effect on patient prognosis (Fig. 3C and D).

**Table 2:** The prognostic IRGPs and their risk coefficients involved in PRSM

IRG 1	Immune-related function	IRG 2	Immune-related function	Coef.
BTLA	Negatively regulating antigen receptor signaling	GPR174	Putative receptor for purines coupled to G-proteins	0.068936
CD244	Regulating innate and adaptive immune response	GPR174	Putative receptor for purines coupled to G-proteins	0.311926
CD28	T cell activation	FASLG	Termination of immune responses	0.279403
CD80	T-lymphocyte activation	RGS18	Inhibiting signal transduction	− 0.32299
CERKL	Regulate autophagy	RHOH	Negative regulator of hematopoietic progenitor cell	− 0.14509
CLEC4A	Regulating immune reactivity	LILRB4	Down-regulation of the immune response and the development of tolerance	0.065819
CYTH4	Activation of ARF factors	STAC3	–	− 0.33071
EVI2B	Hematopoietic progenitor cells differentiation	SLAMF7	Regulating innate and adaptive immune response	− 0.41131
FCRL3	B-cell proliferation	LY9	Negative regulator of the immune response	− 0.13661
FGR	Regulation of immune responses (including neutrophil, monocyte, macrophage and mast cell functions)	IL10RA	Participating in IL10-mediated anti-inflammatory functions	0.386338
FOXP3	The development and inhibitory function of regulatory T-cells (Treg)	RASAL3	Playing an important role in the expansion and functions of natural killer T (NKT) cells	− 0.42278
FUT7	Involved in cell and matrix adhesion during leukocyte trafficking and fertilization	TFEC	Transcriptional regulator	0.190542
GPR34	Orphan receptor	STAC3	–	0.345396
GYPC	Regulating the stability of red cells	LST1	Involved in dendritic cell maturation	0.097751
IGSF6	Regulating the function of CD8 + T effector cells	TNFSF13B	Involved in the stimulation of B- and T-cell function	0.058717
LCP2	Involved in T-cell antigen receptor mediated signaling	SLAMF8	B-lineage commitment and/or modulation of signaling	0.241523
LY9	Negative regulator of the immune response	ZC3H12D	Degradation of interleukin IL-6 mRNA level in activated macrophages	0.250874
NLRC3	Negative regulator of the innate immune response	SIGLEC1	Macrophage-restricted adhesion molecule	0.005176
SAMD9L	Involved in endosome fusion	TMEM140	Inhibition of viral proliferation	− 0.18399

### Prognosis-related immune infiltrating cells

**Fig. 4:**
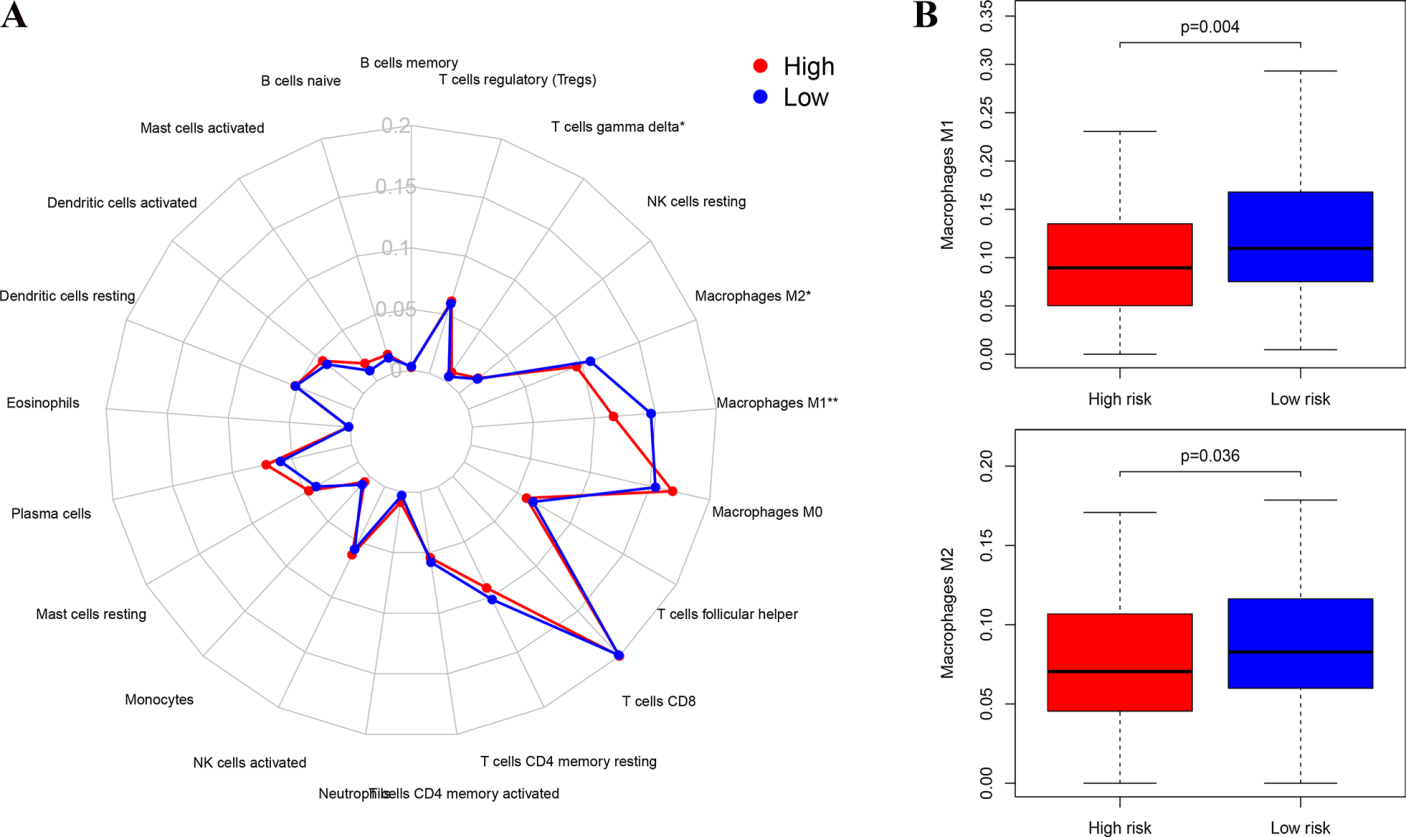
Exploration of the differences in immune infiltration. **A** Summary of the 22 immune cells infiltration abundance in the different risk groups. **B** The difference analysis of the content of various immune cells in the high and low risk group. Macrophage M1 (p = 0.004) and M2 (p = 0.036) were significantly highly expressed in the low-risk group

To analyze the differences in immune infiltration among the high- and low-risk groups, we used CIBERSORT to estimate the relative infiltration abundance of 22 immune cells in the different samples. A difference analysis of the content of various immune cells in the high and low risk group revealed the high level of macrophage (M1 and M2) infiltration in the low-risk group (Fig. 4A and B). This finding suggests that differences in macrophage infiltration may be an important factor affecting the prognosis of CC.

### Functional enrichment analysis and identification of key genes

**Fig. 5:**
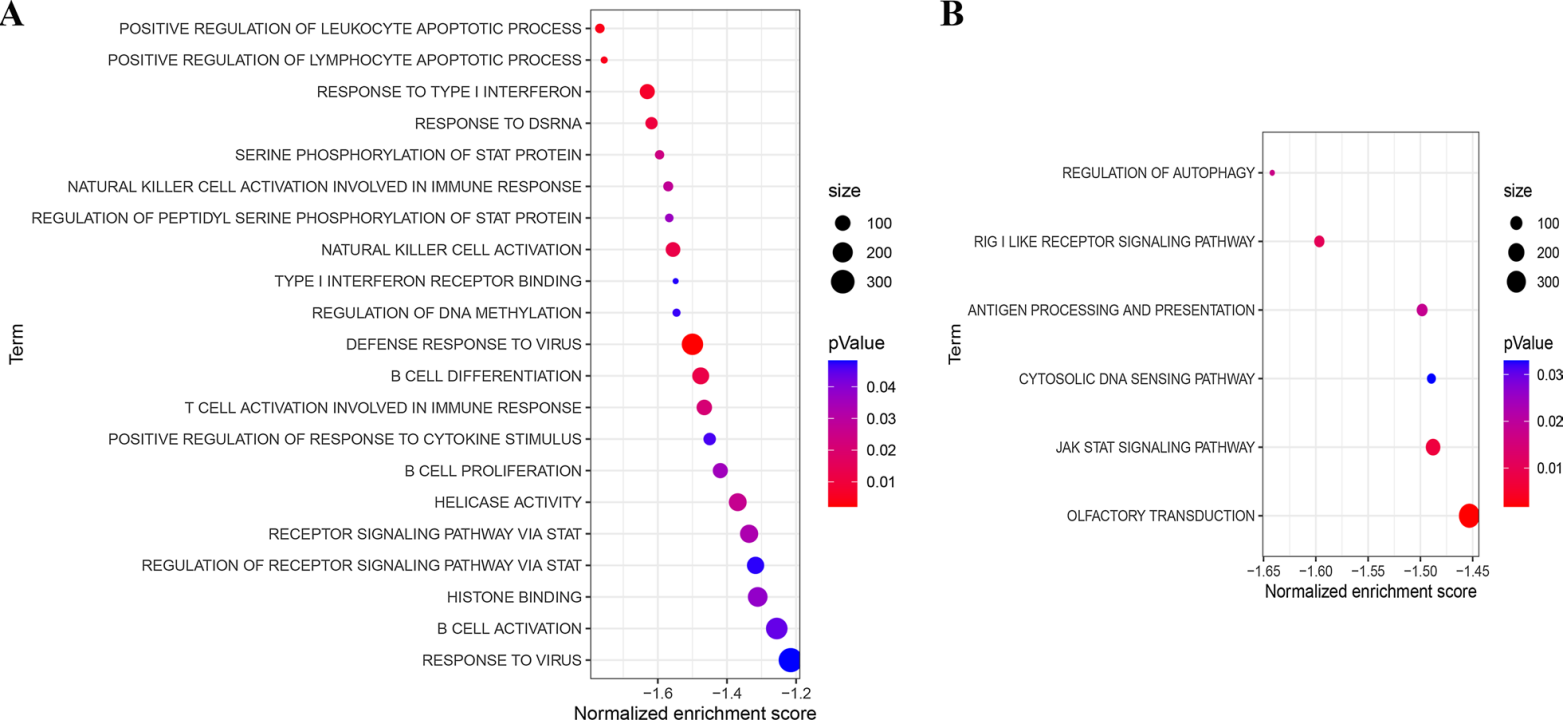
The biological functions and signaling pathways in the high and low-risk group. **A** Gene Ontology (GO) analysis of biological functions. **B** Kyoto Encyclopedia of Genes and Genomes (KEGG) analysis of signaling pathways

To investigate the differences in biological functions and signaling pathways in the high and low-risk group, we performed a GO (Fig. 5A) and KEGG (Fig. 5B)-related GSEA. The results showed that retinoic acid-inducible gene-I (RIG-I)-like receptors (RLRs) and the Janus kinase-signal transducer and activator of transcription (JAK-STAT) signaling pathway were significantly enriched in the low-risk group, and most of these pathways are involved in immune activation (Fig. 5A and B). These results provided molecular implications for differences in immune infiltration and prognosis in the high- and low-risk groups.

**Fig. 6:**
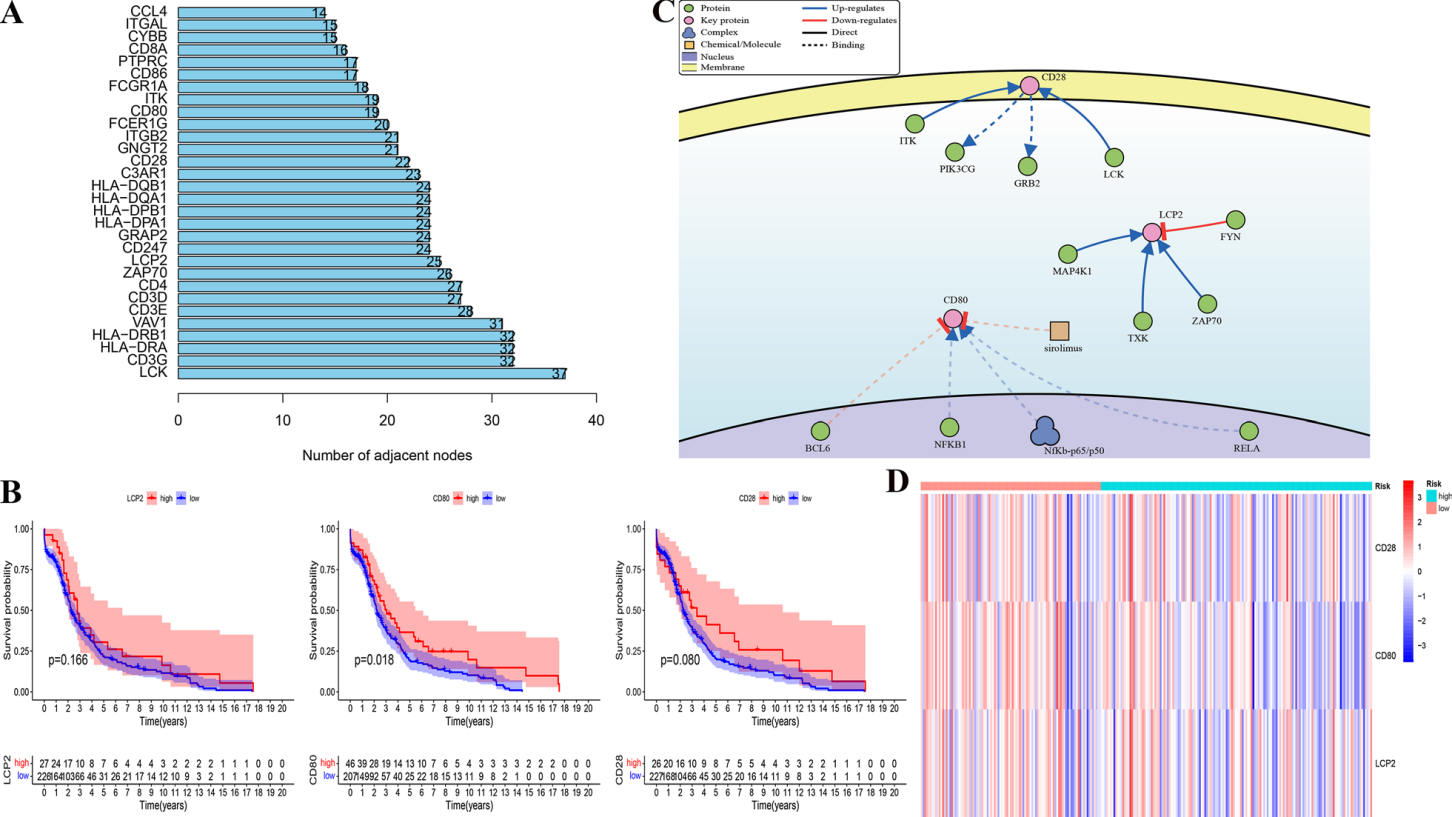
Identification of Key IRGs. **A** The Top 30 hub genes in the protein–protein interaction (PPI) analyses. **B** Kaplan–Meier curves of overall survival in three prognostic key IRGs. **C** The network of intersects genes in DisNor. **D** The heatmap of 3 key IRGs expression patterns with risk group

**Fig. 7:**
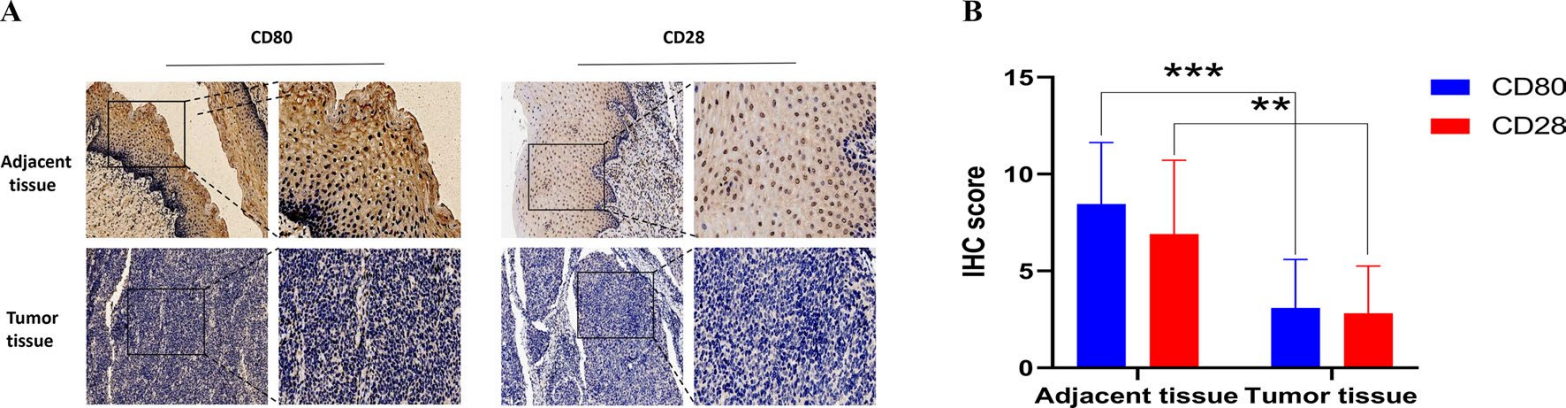
IHC of CD80 and CD28 in adjacent normal tissues and tumor tissues from patients with cervical cancer. **A** CD80 and CD28 expression is lower in cervical squamous cell carcinoma tissues compared with adjacent normal tissues. **B** Comparison of CD80 and CD28 protein expression in 11 pairs of matched tissue section samples by IHC

To facilitate the clinical application of novel prognostic biomarkers, we further identified the key prognostic immune genes in CC. By constructing the protein interaction network of 301 DEGs obtained above, we calculated the number of adjacent nodes for each gene (Fig. 6A). A greater number of nodes indicated a more important role in the network. We screened genes with a current node count of 30 and intersected them with the genes contained in the PRSM. Finally, we obtained three key immune-related genes: CD80, CD28, and LCP2. The prognostic curve of these three genes was plotted and it was found that the prognosis of the high expression group was more favorable compared to that of the low expression group (Fig. 6B). We identified the genes directly responsible for these key genes using the DisNor database. NFKB1, NFKB-p65 /p50, and RelA are located upstream of CD80. ITK and LCK are upstream, while PIK3CG and GRB2 are located downstream of CD28. MAP4K1, TXK, and ZAP70 are upstream of LCP2 (Fig. 6C). The expressions of the three key IRGs in the high and low risk score groups was shown using a heatmap (Fig. 6D). The genes with the significant prognosis were extracted for further analysis. To further verify the expression of CD80, CD28 at the tissue level. By testing 11 cervical cancer tissue and 11 adjacent normal tissues specimens using TMA, CD80 and CD28 expression are lower in cervical squamous cell carcinoma tissues (Fig. 7).

### Sensitivity analysis of therapy

**Fig. 8:**
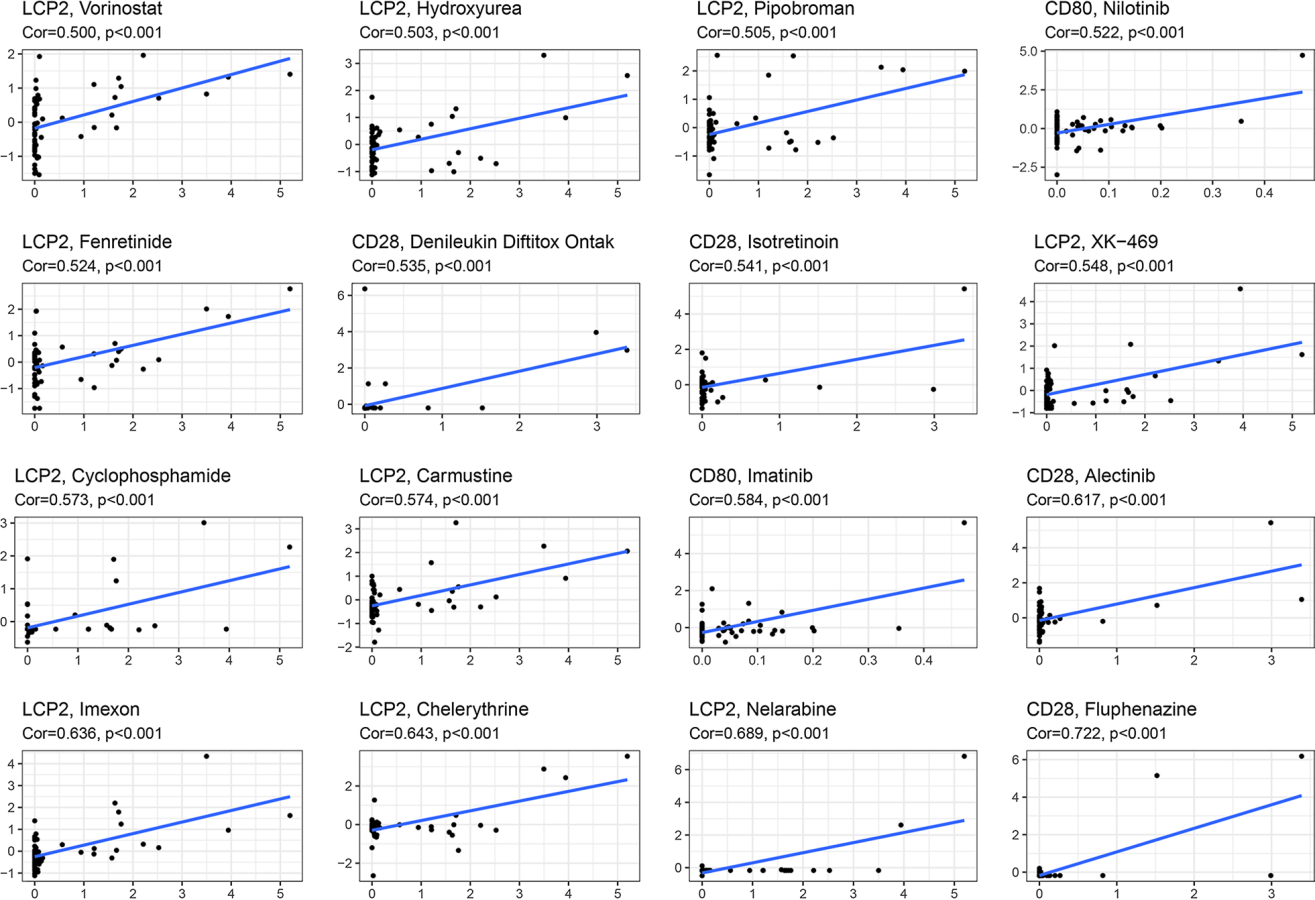
The chemotherapy drug sensitivity analysis of the three key genes. The level of the three key IRGs expression was positively correlated with drug sensitivity

The chemotherapy drug sensitivity analysis of the three key genes revealed that the level of gene expression was positively correlated with drug sensitivity to Vorinotat, Cyclophosphamide, Nilotinib, and Imatinib, etc. (Fig. 8).

**Fig. 9:**
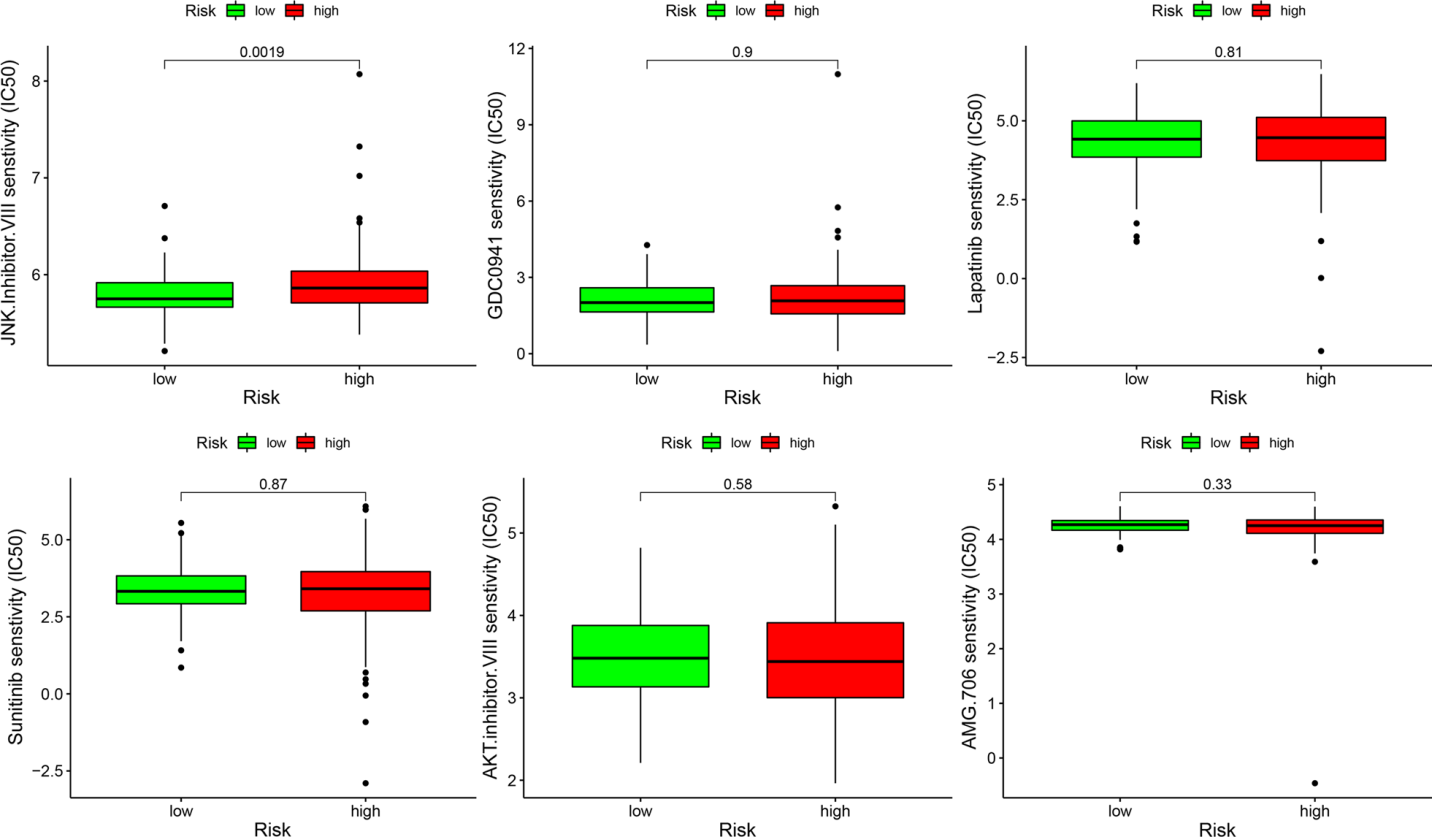
Potential drugs for the treatment of cervical cancer. The sensitivity of multiple target drugs in different risk groups

Next, we analyzed and predicted the sensitivity of multiple target drugs in different risk groups to screen out potential drugs for the treatment of cervical cancer. Finally, we found that differences in sensitivity to JNK inhibitors could be distinguished by the use and risk grouping of this PRSM (Fig. 9). These results indicate that the PRSM and key immune genes have great significance for clinical treatment, and can be used to identify targeted drugs that can be affected by immune factors.

## Discussion

Recently, immunotherapy has opened a new door as a novel treatment method for CC. However, the efficacy has not been impressive [18]. The tumor immune microenvironment plays an important role in tumor progression. Substantial evidences show that IRGs are associated with the prognosis of patients with multiple solid tumors [19, 20]. Moreover, there is evidence that immune-related signals can predict the prognosis of CC [21]. Therefore, it is of great significance to identify immune-related models for the prognosis of patients with CC and improve their clinical benefits.

In this study, we use the ESTIMATION algorithm calculate the score of the immune cells or stromal cells and divide them into high and low scores. The results show a strong correlation between these scores and survival in patients with CC. Moreover, to eliminate measurement errors among different samples, we constructed the model using gene pairs. Prognostic models show a superior ability to classify patients into low- and high-risk groups. We identified that patients in the low-risk group showed favorable outcomes, and there is supporting evidence that our model can stratify risk. Moreover, we analyzed the immune cells in different risk groups, and found that there was obvious macrophage infiltration in the low-risk group. Macrophages are one of the most important immune cells. Monocytes can differentiate into two different types of macrophages: M1 (proinflammatory, classically activated macrophages) and M2 (anti-inflammatory, alternatively activated macrophages) phenotypes [22]. M2 macrophages can be further refined into M2a, M2b, M2c, and M2d subsets [23]. The above evidence shows that differences in immune infiltration are related to the prognosis of CC patients. However, macrophage polarization is a complex process in the immune microenvironment. The relationship between specific phenotypic differences in polarization and prognosis requires further analysis.

In cancer immunotherapy, the activation of retinoic acid-inducible gene-I (RIG-I)-like receptors (RLRs) can induce anti-tumor effects in various cancers [24]. RIG-I agonist therapy can enhance the activity of anti-cancer effector cells (e.g., cytotoxic T cells and NK cells) and block the activity of immunosuppressive cells (e.g., regulatory T cells and myeloid-derived suppressor cells [MDSCs]), successfully induce the killing of tumor cells, and regulate the TME [25]. JAK-STAT signaling can mediate the majority of the immune-modulatory processes, including tumor recognition and immune escape [26]. The JAK-STAT signaling pathway is a double-edged sword in CIT, and both STAT1 and STAT2 drive anti-tumor immune responses by inducing type I and type II interferon (IFN) [27]. In contrast, STAT3 is widely associated with immunosuppression, cancer cell survival, and persistent inflammation in the tumor microenvironment [28, 29]. Studies have shown that the JAK-STAT signaling pathway can interact with Toll-like receptors (TLRs), co-regulate the M1 macrophage polarization, and the inflammatory response of macrophages [30]. The JAK/STAT signaling pathway can also be used as a novel tumor marker and prognostic factor for the diagnosis and prognosis of CC [31]. Our results provide evidence that RLRs and the JAK/STAT signaling pathway are associated with patient prognosis.

We verified that three representative genes (CD80, CD28, and LCP2) were markers of CC prognosis. CD80 and CD28 may more prominent represent important indicators to improve patient prognosis after validated by immunohistochemistry. CD80 is one of the most potent costimulatory molecules involved in tumor cell recognition and killing, is activated by CD28 or CTLA-4 binding, and can induce T cell proliferation and cytokine production [32]. TLRs activate the adaptive immune response by producing pro-inflammatory cytokines and inducing key surface molecules (e.g., CD80) [33]. In colon cancer, low CD80 expression is associated with immune escape and plays an important role in the immune surveillance of the lesion [34]. In breast cancer, CD80 may lead to the progression and metastasis of breast cancer by regulating the innate immune system [35]. CD28 represents one of many proteins, defining a subfamily of costimulatory receptors and ligands [36]. CD28 optimizes the T cell response during antigen recognition by enhancing TCR signaling or other unique signaling pathways [37, 38]. In addition, the majority of the proliferating CD8 + T cells in NSCLC patients receiving anti-PD-1 treatment were found to be CD28-positive [39]. In addition, our findings confirm that CD28 bispecific antibodies can enhance the anti-tumor efficacy of treatment with CD3 bispecific antibodies [40]. CD28 is also associated with the prognosis of patients with prostate cancer [41]. LCP2 encodes a protein containing 533 amino acids that is involved in T cell activation and can increase the IL-2 gene promoter activity following transient overexpression [42]. High LCP2 expression is associated with a better prognosis in patients with diffuse large B cell lymphoma [43]. Therefore, the key prognostic IRGs have potential clinical significance for predicting survival. We used the model to test the therapeutic sensitivity and screen for sensitive drugs based on the key genes. High-risk cervical cancer patients are more sensitive to treatment with JNK inhibitors. Thus, the predictive model and key prognostic IRGs are also valuable for the clinical treatment of CC.

In conclusion, our study establishes a novel prognostic model by selecting DEGs in immune and stromal cells, and three IRGs were identified to evaluate patient prognosis. Moreover, CD80 and CD28 may more prominent represent important indicators to improve patient prognosis. In addition, the analysis of relevant pathways and sensitivity to molecular chemotherapy and molecular therapy can help select drugs with high clinical benefit and provide a basis for the precise treatment of CC. Of course, these immune-prognostic markers for the diagnosis, prognosis, and treatment of CC require further clinical confirmation.

## Supplementary Information

**Additional file 1: Table S1.** The immune score and stromal score of CC samples. **Table S2.** The IRGPs with significant prognostic differences in univariate Cox proportional hazards regression analysis.

## Data Availability

The datasets supporting the conclusions of this article are included within the article.

## Abbreviations

CC: Cervical cancer
PRSM: Prognostic risk scoring model
IRGs: Immune-related genes
DEGs: Differentially expressed genes
WGCNA: Weighted gene co-expression network analysis
IRGPs: Immune-related gene pairs
PPIs: Protein–protein interactions
RLRs: Retinoic acid-inducible gene-I (RIG-I)-like receptors
JAK-STAT: Janus kinase-signal transducer and activator of transcription
OS: Overall survival
TME: Tumor microenvironment
RS: Risk score
Tis: Targeted inhibitors
KEGG: Kyoto encyclopedia of genes and genomes
GSEA: Gene set enrichment analysis
IHC: Immunohistochemistry
TMA: Tissue microarrays assay
TLRs: Toll-like receptors
