# Supplementary material for: Comprehensive analysis of prognostic immune-related genes and drug sensitivity in cervical cancer
Source: Cancer Cell Int. 2021 Dec 1;21:639. doi: 10.1186/s12935-021-02333-9 (PMC8638517; doi:10.1186/s12935-021-02333-9)
Supplement: Supplementary file 1 — Additional file 1: Table S1. The immune score and stromal score of CC samples. Table S2. The IRGPs with significant prognostic differences in univariate Cox proportional hazards regression analysis. [file 12935_2021_2333_MOESM1_ESM.docx]

Table S1. The immune score and stromal score of CC samples.

| ID | Stromal Score | Immune Score | ESTIMATE  Score |
| --- | --- | --- | --- |
| TCGA-C5-A7UC-01A-11R-A352-07 | -1490 | -354.945 | -1844.94 |
| TCGA-EA-A3QD-01A-32R-A22U-07 | 329.465 | 2621.016 | 2950.481 |
| TCGA-C5-A901-01A-11R-A37O-07 | -731.809 | 635.2797 | -96.5295 |
| TCGA-C5-A8YT-01A-11R-A37O-07 | 37.99946 | 330.5328 | 368.5322 |
| TCGA-EK-A2PM-01A-11R-A18M-07 | -1349.63 | -1109.9 | -2459.53 |
| TCGA-EK-A2RB-01A-11R-A18M-07 | -1408.35 | 474.4195 | -933.927 |
| TCGA-VS-A9UM-01A-11R-A42T-07 | -187.49 | 1331.299 | 1143.808 |
| TCGA-VS-A9V2-01A-11R-A42T-07 | -811.396 | 1463.044 | 651.6481 |
| TCGA-C5-A907-01A-11R-A37O-07 | -1068.52 | 993.4356 | -75.0806 |
| TCGA-MY-A5BD-01A-11R-A26T-07 | -1736.82 | -55.7033 | -1792.52 |
| TCGA-FU-A3HZ-01A-11R-A213-07 | -1056.52 | 174.0042 | -882.519 |
| TCGA-C5-A1MH-01A-11R-A14Y-07 | -6.42425 | 1980.61 | 1974.186 |
| TCGA-IR-A3L7-01A-21R-A213-07 | -1296.94 | -452.959 | -1749.9 |
| TCGA-MA-AA3W-01A-11R-A38B-07 | -832.37 | 2278.879 | 1446.509 |
| TCGA-C5-A8XI-01A-11R-A37O-07 | -1033.48 | 1084.275 | 50.79438 |
| TCGA-MA-AA3X-01A-22R-A42S-07 | -311.125 | 1336.928 | 1025.802 |
| TCGA-VS-AA62-01A-11R-A42T-07 | -457.039 | 2026.154 | 1569.116 |
| TCGA-C5-A1BF-01B-11R-A13Y-07 | -281.153 | 1087.914 | 806.7611 |
| TCGA-C5-A1BI-01B-11R-A13Y-07 | -280.965 | 1672.525 | 1391.559 |
| TCGA-HM-A6W2-06A-22R-A33Z-07 | -1593.64 | -731.179 | -2324.81 |
| TCGA-FU-A3TX-01A-11R-A22U-07 | -1132.03 | 860.8318 | -271.2 |
| TCGA-C5-A2LZ-01A-11R-A213-07 | -1433.11 | 974.2072 | -458.905 |
| TCGA-ZJ-A8QR-01A-11R-A37O-07 | -1467.74 | 506.8197 | -960.922 |
| TCGA-JW-A5VG-01A-11R-A28H-07 | -1266.59 | 1199.759 | -66.8336 |
| TCGA-MU-A5YI-01A-11R-A32P-07 | 365.0466 | 1069.41 | 1434.456 |
| TCGA-EA-A410-01A-11R-A32Y-07 | -275.697 | 232.7237 | -42.9738 |
| TCGA-JX-A3PZ-01A-11R-A32Y-07 | -353.686 | 1473.333 | 1119.647 |
| TCGA-EX-A69L-01A-11R-A32P-07 | -304.217 | 2133.342 | 1829.125 |
| TCGA-VS-A953-01A-11R-A38B-07 | -1629.5 | 487.9031 | -1141.6 |
| TCGA-C5-A1M5-01A-11R-A13Y-07 | -873.771 | 1345.755 | 471.9842 |
| TCGA-C5-A902-01A-11R-A37O-07 | -1443.61 | 1513.138 | 69.53064 |
| TCGA-C5-A1M7-01A-11R-A13Y-07 | -852.09 | 1150.467 | 298.3767 |
| TCGA-DG-A2KK-01A-11R-A180-07 | -1232.37 | 688.2106 | -544.161 |
| TCGA-EA-A3HU-01A-11R-A213-07 | -527.747 | 2259.583 | 1731.836 |
| TCGA-C5-A3HD-01B-11R-A213-07 | -2049.26 | -248.014 | -2297.27 |
| TCGA-EA-A3HQ-01A-11R-A213-07 | -397.998 | 1352.473 | 954.4749 |
| TCGA-EX-A8YF-01A-11R-A37O-07 | -1010.29 | 1473.632 | 463.3406 |
| TCGA-DS-A0VN-01A-21R-A10U-07 | -79.1473 | 2147.426 | 2068.279 |
| TCGA-EK-A2H0-01A-11R-A180-07 | -1777.53 | 496.6659 | -1280.86 |
| TCGA-DS-A5RQ-01A-11R-A28H-07 | -913.602 | 1096.437 | 182.8344 |
| TCGA-Q1-A6DT-01A-11R-A32P-07 | -1248.8 | 75.67971 | -1173.12 |
| TCGA-ZJ-AAXA-01A-11R-A42T-07 | -931.392 | -55.5327 | -986.925 |
| TCGA-EA-A1QT-01A-11R-A14Y-07 | -1077.86 | 424.7543 | -653.111 |
| TCGA-MY-A5BE-01A-21R-A26T-07 | 53.18107 | 3143.154 | 3196.335 |
| TCGA-MU-A51Y-01A-11R-A26T-07 | -138.918 | 1931.063 | 1792.145 |
| TCGA-FU-A23K-01A-11R-A16R-07 | -387.718 | 1481.653 | 1093.934 |
| TCGA-EA-A3HR-01A-11R-A213-07 | -1044.67 | 282.5206 | -762.145 |
| TCGA-VS-A8QC-01A-11R-A37O-07 | -500.527 | 389.7619 | -110.765 |
| TCGA-ZJ-AB0I-01A-11R-A42T-07 | -1121.62 | 634.6209 | -487 |
| TCGA-GH-A9DA-01A-21R-A37O-07 | -785.071 | 1013.826 | 228.7548 |
| TCGA-EK-A2RC-01A-11R-A18M-07 | -346.748 | 1472.056 | 1125.308 |
| TCGA-EA-A44S-01A-12R-A26T-07 | -749.403 | 1123.612 | 374.2091 |
| TCGA-Q1-A6DW-01A-11R-A32P-07 | -996.541 | 535.7637 | -460.777 |
| TCGA-FU-A2QG-01A-11R-A18M-07 | -1781.88 | 474.4264 | -1307.45 |
| TCGA-C5-A1MK-01A-11R-A14Y-07 | 80.84922 | 700.3404 | 781.1896 |
| TCGA-ZJ-A8QO-01A-11R-A37O-07 | -157.636 | 1650.984 | 1493.349 |
| TCGA-MA-AA43-01A-11R-A42T-07 | -356.355 | 177.4884 | -178.867 |
| TCGA-C5-A1BL-01A-11R-A13Y-07 | -1222.85 | 1268.416 | 45.56427 |
| TCGA-ZJ-A8QQ-01A-11R-A37O-07 | -1520.79 | 415.5765 | -1105.22 |
| TCGA-VS-A8EJ-01A-11R-A37O-07 | -1266.64 | 297.2082 | -969.429 |
| TCGA-C5-A7UI-01A-11R-A36F-07 | -678.7 | 1697.245 | 1018.545 |
| TCGA-Q1-A73Q-01A-21R-A32P-07 | -1376.17 | 27.35966 | -1348.81 |
| TCGA-DS-A1OA-01A-11R-A14Y-07 | -448.953 | 1396.824 | 947.8705 |
| TCGA-C5-A8XK-01A-11R-A37O-07 | -1292.27 | 485.3136 | -806.959 |
| TCGA-C5-A905-01A-11R-A37O-07 | -1825.25 | 98.50516 | -1726.74 |
| TCGA-C5-A7X5-01A-11R-A36F-07 | -1301.63 | -432.473 | -1734.1 |
| TCGA-EK-A2RM-01A-21R-A18M-07 | -346.924 | 76.75171 | -270.172 |
| TCGA-IR-A3LL-01A-11R-A213-07 | -573.266 | 2202.125 | 1628.859 |
| TCGA-DR-A0ZM-01A-12R-A10U-07 | -355.438 | 1920.016 | 1564.578 |
| TCGA-R2-A69V-01A-11R-A32P-07 | 226.7028 | 2995.468 | 3222.171 |
| TCGA-C5-A7UH-01A-11R-A352-07 | -833.472 | 622.1662 | -211.306 |
| TCGA-VS-A9UH-01A-11R-A42T-07 | 340.0476 | 2347.258 | 2687.306 |
| TCGA-LP-A4AV-01A-11R-A32Y-07 | -125.595 | 1752.991 | 1627.396 |
| TCGA-VS-A8QF-01A-21R-A37O-07 | -701.795 | 1117.324 | 415.5282 |
| TCGA-UC-A7PG-01A-11R-A42S-07 | -807.251 | 237.903 | -569.348 |
| TCGA-C5-A0TN-01A-21R-A14Y-07 | -1123.71 | 523.1679 | -600.539 |
| TCGA-C5-A1MQ-01A-11R-A14Y-07 | 43.17585 | 2048.85 | 2092.026 |
| TCGA-EX-A69M-01A-11R-A32P-07 | -1132.74 | 1676.264 | 543.5205 |
| TCGA-C5-A1MP-01A-11R-A14Y-07 | 321.5071 | 1943.492 | 2264.999 |
| TCGA-C5-A7CG-01A-11R-A32P-07 | -397.351 | 2633.145 | 2235.793 |
| TCGA-C5-A1BN-01B-11R-A14Y-07 | -1717.78 | -330.419 | -2048.2 |
| TCGA-FU-A5XV-01A-11R-A28H-07 | -1222.5 | 381.2196 | -841.279 |
| TCGA-EA-A3QE-01A-21R-A21T-07 | -562.431 | 2162.405 | 1599.974 |
| TCGA-VS-A8Q8-01A-11R-A37O-07 | -843.549 | 1458.523 | 614.9741 |
| TCGA-BI-A0VR-01A-11R-A10U-07 | -510.783 | 1628.238 | 1117.455 |
| TCGA-EK-A2PL-01A-11R-A18M-07 | -1522.37 | 384.8437 | -1137.53 |
| TCGA-DR-A0ZL-01A-11R-A10U-07 | -1775.08 | 36.80365 | -1738.28 |
| TCGA-LP-A5U3-01A-11R-A28H-07 | -1218.19 | 976.4161 | -241.774 |
| TCGA-MY-A5BF-01A-11R-A26T-07 | -1058.6 | 1342.693 | 284.0956 |
| TCGA-JW-A5VH-01A-11R-A28H-07 | -815.086 | -948.256 | -1763.34 |
| TCGA-C5-A1BK-01B-11R-A13Y-07 | -544.345 | 2345.604 | 1801.259 |
| TCGA-VS-A8EC-01A-11R-A36F-07 | -2092.67 | -1169.64 | -3262.31 |
| TCGA-IR-A3LH-01A-21R-A213-07 | 626.5818 | 2626.364 | 3252.946 |
| TCGA-VS-A8QA-01A-11R-A37O-07 | -2064.66 | 396.2305 | -1668.43 |
| TCGA-C5-A8XJ-01A-11R-A37O-07 | -242.792 | 416.4738 | 173.6816 |
| TCGA-EA-A5FO-01A-21R-A28H-07 | -2318.31 | -538.609 | -2856.92 |
| TCGA-FU-A3HY-01A-11R-A21T-07 | -1718.55 | -159.579 | -1878.13 |
| TCGA-FU-A3TQ-01A-11R-A22U-07 | -779.764 | 968.4304 | 188.6668 |
| TCGA-VS-A94X-01A-11R-A38B-07 | -1530.71 | 421.8005 | -1108.91 |
| TCGA-EA-A5O9-01A-11R-A28H-07 | -1418 | 674.6693 | -743.333 |
| TCGA-VS-A9U5-01A-11R-A42T-07 | -1325.09 | 1183.528 | -141.56 |
| TCGA-EK-A2IP-01A-11R-A180-07 | -1468.68 | 428.0588 | -1040.62 |
| TCGA-RA-A741-01A-11R-A33Z-07 | -613.406 | 1594.035 | 980.6294 |
| TCGA-IR-A3LC-01A-11R-A213-07 | -1083.7 | 619.9156 | -463.784 |
| TCGA-DG-A2KM-01A-11R-A180-07 | 582.4582 | 3419.222 | 4001.68 |
| TCGA-FU-A3YQ-01A-11R-A22U-07 | -6.62356 | 1825.642 | 1819.019 |
| TCGA-EK-A3GN-01A-11R-A213-07 | -1967.02 | -507.327 | -2474.34 |
| TCGA-C5-A8XH-01A-11R-A37O-07 | -949.323 | 1127.579 | 178.2564 |
| TCGA-C5-A1BM-01A-11R-A13Y-07 | -376.183 | 1803.271 | 1427.088 |
| TCGA-DS-A1OB-01A-11R-A14Y-07 | -805.067 | 745.3313 | -59.7359 |
| TCGA-EA-A43B-01A-81R-A32Y-07 | -549.218 | 1487.775 | 938.5577 |
| TCGA-BI-A20A-01A-11R-A14Y-07 | -622.843 | 1567.721 | 944.8782 |
| TCGA-MA-AA41-01A-11R-A38B-07 | -1356.67 | 544.6296 | -812.037 |
| TCGA-EA-A411-01A-11R-A24H-07 | 174.2962 | 654.4778 | 828.7739 |
| TCGA-DS-A3LQ-01A-21R-A21T-07 | -45.0606 | 579.581 | 534.5203 |
| TCGA-EK-A2PK-01A-11R-A18M-07 | -323.589 | 2038.173 | 1714.584 |
| TCGA-VS-A950-01A-11R-A42T-07 | -930.825 | 327.6239 | -603.201 |
| TCGA-VS-A94W-01A-12R-A37O-07 | -649.767 | 1824.11 | 1174.343 |
| TCGA-VS-A959-01A-11R-A42T-07 | -860.279 | 1145.571 | 285.2917 |
| TCGA-EX-A1H5-01A-31R-A13Y-07 | -1267.36 | 104.144 | -1163.22 |
| TCGA-Q1-A5R2-01A-11R-A28H-07 | -452.803 | 1532.041 | 1079.238 |
| TCGA-HM-A3JK-01A-11R-A32Y-07 | 640.533 | 1134.408 | 1774.941 |
| TCGA-C5-A7CJ-01A-11R-A32P-07 | 157.2159 | 1555.832 | 1713.048 |
| TCGA-EK-A2PG-01A-11R-A18M-07 | -2031.16 | 29.00222 | -2002.16 |
| TCGA-VS-A957-01A-11R-A42T-07 | -2081.69 | -64.394 | -2146.08 |
| TCGA-HG-A2PA-01A-11R-A213-07 | -620.934 | 899.5875 | 278.6533 |
| TCGA-MA-AA3Y-01A-11R-A38B-07 | -438.01 | 1755.149 | 1317.139 |
| TCGA-VS-A9UI-01A-11R-A42T-07 | -1309.78 | 1209.471 | -100.314 |
| TCGA-VS-A94Y-01A-11R-A38B-07 | -710.247 | 586.7196 | -123.528 |
| TCGA-C5-A2LV-01A-11R-A18M-07 | -643.92 | 1396.712 | 752.7917 |
| TCGA-C5-A8YR-01A-12R-A37O-07 | -732.356 | 1508.482 | 776.1262 |
| TCGA-JW-A852-01A-11R-A352-07 | -1446.44 | 770.4173 | -676.025 |
| TCGA-VS-A9UJ-01A-11R-A42T-07 | -1514.41 | -995.714 | -2510.12 |
| TCGA-DS-A0VK-01A-21R-A10U-07 | -795.311 | 70.09402 | -725.217 |
| TCGA-DG-A2KL-01A-11R-A180-07 | 463.4344 | 1076.897 | 1540.332 |
| TCGA-DG-A2KJ-01A-11R-A32Y-07 | -672.884 | 1798.215 | 1125.332 |
| TCGA-DS-A7WI-01A-12R-A352-07 | -1430.81 | 865.2282 | -565.585 |
| TCGA-C5-A7CO-01A-11R-A352-07 | -1472.72 | 595.9913 | -876.73 |
| TCGA-ZJ-AAX8-01A-11R-A42T-07 | 282.4673 | 1434.158 | 1716.625 |
| TCGA-LP-A4AU-01A-32R-A32Y-07 | -920.349 | 804.6787 | -115.67 |
| TCGA-C5-A3HE-01A-21R-A22U-07 | -730.288 | 1664.835 | 934.5478 |
| TCGA-EK-A2R8-01A-21R-A18M-07 | -1025.82 | 458.3072 | -567.51 |
| TCGA-UC-A7PD-01A-11R-A352-07 | -1291.51 | 321.7064 | -969.806 |
| TCGA-MA-AA3Z-01A-11R-A38B-07 | -1534.45 | 1247.971 | -286.478 |
| TCGA-JW-A5VK-01A-11R-A28H-07 | -1691.29 | -105.648 | -1796.94 |
| TCGA-DS-A1O9-01A-11R-A14Y-07 | 15.90664 | 1699.319 | 1715.226 |
| TCGA-IR-A3LK-01A-12R-A213-07 | -1371.96 | 1024.127 | -347.837 |
| TCGA-EA-A50E-01A-21R-A26T-07 | 301.9648 | 788.9547 | 1090.92 |
| TCGA-EK-A2RE-01A-11R-A18M-07 | -1308.85 | -341.785 | -1650.63 |
| TCGA-ZJ-AAXD-01A-21R-A42T-07 | -551.746 | 217.9029 | -333.843 |
| TCGA-ZJ-AAXN-01A-11R-A42T-07 | -817.707 | 1035.662 | 217.9552 |
| TCGA-BI-A0VS-01A-11R-A10U-07 | -503.377 | 860.0729 | 356.6956 |
| TCGA-C5-A1ML-01A-11R-A14Y-07 | -1010.58 | 613.9667 | -396.612 |
| TCGA-MU-A8JM-01A-11R-A36F-07 | -958.894 | 1106.228 | 147.3339 |
| TCGA-EK-A2RJ-01A-11R-A18M-07 | -749.268 | 1505.562 | 756.2944 |
| TCGA-DS-A0VL-01A-21R-A10U-07 | -1156.25 | 956.8988 | -199.355 |
| TCGA-4J-AA1J-01A-21R-A38B-07 | -692.311 | 86.05203 | -606.259 |
| TCGA-FU-A23L-01A-11R-A16R-07 | -958.826 | 1552.485 | 593.6582 |
| TCGA-ZJ-AAXI-01A-11R-A42T-07 | -1611.08 | 409.8451 | -1201.24 |
| TCGA-EK-A2R9-01A-11R-A18M-07 | -68.0732 | 1411.525 | 1343.452 |
| TCGA-JW-A5VL-01A-11R-A28H-07 | -1011.61 | 1491.769 | 480.162 |
| TCGA-VS-A8EI-01A-11R-A37O-07 | -731.615 | 1209.194 | 477.5797 |
| TCGA-EA-A97N-01A-11R-A38B-07 | -293.385 | 248.0695 | -45.3158 |
| TCGA-EA-A78R-01A-11R-A32P-07 | -298.526 | 1097.038 | 798.512 |
| TCGA-C5-A7CK-01A-11R-A32P-07 | -514.471 | -522.684 | -1037.15 |
| TCGA-FU-A3NI-01A-11R-A21T-07 | -642.201 | 580.4324 | -61.769 |
| TCGA-C5-A8YQ-01A-11R-A37O-07 | -1196.2 | 489.5005 | -706.698 |
| TCGA-VS-A9U6-01A-11R-A42T-07 | -744.05 | 992.2731 | 248.2233 |
| TCGA-VS-A94Z-01A-11R-A38B-07 | -366.002 | 2240.319 | 1874.316 |
| TCGA-C5-A1BE-01B-11R-A13Y-07 | -994.645 | 534.0082 | -460.637 |
| TCGA-WL-A834-01A-11R-A352-07 | -1030.35 | -105.906 | -1136.26 |
| TCGA-Q1-A73O-01A-11R-A32P-07 | -871.816 | 1000.421 | 128.6054 |
| TCGA-JW-AAVH-01A-11R-A38B-07 | -1770.1 | 56.30774 | -1713.79 |
| TCGA-HM-A4S6-01A-11R-A26T-07 | -263.507 | 1598.341 | 1334.834 |
| TCGA-VS-A9UV-01A-11R-A42T-07 | -1539.64 | 150.0698 | -1389.57 |
| TCGA-EK-A2RO-01A-11R-A18M-07 | -225.483 | 337.3664 | 111.883 |
| TCGA-VS-A8EB-01A-11R-A36F-07 | -973.841 | 880.5199 | -93.3216 |
| TCGA-EA-A3Y4-01A-51R-A24H-07 | -605.551 | 2128.515 | 1522.964 |
| TCGA-C5-A1M8-01A-21R-A13Y-07 | -1236.83 | -28.8458 | -1265.68 |
| TCGA-HM-A6W2-01A-21R-A33Z-07 | -372.551 | 296.2715 | -76.2796 |
| TCGA-C5-A7CH-01A-11R-A33Z-07 | -985.954 | 191.3176 | -794.636 |
| TCGA-EK-A2H1-01A-11R-A180-07 | -173.376 | 1531.109 | 1357.734 |
| TCGA-ZJ-AAXT-01A-11R-A42T-07 | -1262.41 | 558.1538 | -704.26 |
| TCGA-EK-A2PI-01A-11R-A18M-07 | -816.976 | -371.727 | -1188.7 |
| TCGA-JX-A5QV-01A-22R-A28H-07 | -1366.82 | 963.9152 | -402.908 |
| TCGA-VS-A9UL-01A-11R-A42T-07 | -1931.48 | -1209.84 | -3141.32 |
| TCGA-PN-A8MA-01A-11R-A36F-07 | -589.804 | 644.0299 | 54.22608 |
| TCGA-EK-A2IR-01A-11R-A180-07 | -1668.5 | -921.052 | -2589.56 |
| TCGA-VS-A9UD-01A-11R-A42T-07 | -971.063 | 2359.148 | 1388.085 |
| TCGA-VS-A954-01A-11R-A38B-07 | -762.301 | 458.4764 | -303.824 |
| TCGA-ZJ-AB0H-01A-11R-A42T-07 | -235.031 | 1776.161 | 1541.129 |
| TCGA-DS-A1OC-01A-11R-A14Y-07 | -1093.41 | 1099.413 | 6.000764 |
| TCGA-VS-A8Q9-01A-12R-A37O-07 | -1683.34 | 254.158 | -1429.18 |
| TCGA-C5-A3HL-01A-11R-A213-07 | -1291.45 | 313.9719 | -977.476 |
| TCGA-DS-A0VM-01A-11R-A10U-07 | -518.916 | 754.9045 | 235.9887 |
| TCGA-EK-A2RN-01A-12R-A213-07 | -946.434 | 1397.82 | 451.3857 |
| TCGA-C5-A2LX-01A-11R-A18M-07 | -288.539 | 2250.827 | 1962.289 |
| TCGA-EA-A5ZE-01A-11R-A28H-07 | -5.62978 | 1609.298 | 1603.668 |
| TCGA-C5-A1BJ-01A-11R-A13Y-07 | -480.466 | 1285.431 | 804.9649 |
| TCGA-C5-A1MN-01A-11R-A14Y-07 | -1391.73 | 217.3523 | -1174.38 |
| TCGA-C5-A7XC-01A-11R-A38B-07 | -454.549 | 1319.61 | 865.0607 |
| TCGA-ZJ-AAXJ-01A-11R-A42T-07 | -1462.53 | 596.3294 | -866.202 |
| TCGA-EA-A1QS-01A-61R-A22U-07 | 803.9545 | 734.4723 | 1538.427 |
| TCGA-C5-A7UE-01A-11R-A33Z-07 | -1561.59 | 414.0775 | -1147.51 |
| TCGA-FU-A3WB-01A-11R-A22U-07 | -1229.99 | -165.881 | -1395.87 |
| TCGA-JW-A5VJ-01A-11R-A28H-07 | -1309.41 | 243.3635 | -1066.04 |
| TCGA-LP-A4AW-01A-11R-A24H-07 | 186.4631 | 362.2182 | 548.6813 |
| TCGA-ZJ-AAXU-01A-11R-A42T-07 | -941.815 | 1520.288 | 578.4732 |
| TCGA-VS-A9UY-01A-11R-A42T-07 | -1042.12 | 916.9872 | -125.13 |
| TCGA-VS-A8EH-01A-11R-A36F-07 | -1661.66 | 25.99788 | -1635.66 |
| TCGA-C5-A1M6-01A-11R-A13Y-07 | -1504.35 | -566.754 | -2071.1 |
| TCGA-EK-A2RA-01A-11R-A18M-07 | -1364.23 | 1691.593 | 327.362 |
| TCGA-VS-A8EG-01A-11R-A36F-07 | -1719.18 | 214.0773 | -1505.1 |
| TCGA-VS-A9V3-01A-11R-A42T-07 | -1008.73 | 1104.303 | 95.57112 |
| TCGA-EA-A5ZF-01A-11R-A28H-07 | -395.456 | -299.438 | -694.894 |
| TCGA-MA-AA42-01A-12R-A38B-07 | -396.717 | 2833.675 | 2436.958 |
| TCGA-VS-A9U7-01A-11R-A42T-07 | -867.552 | 1549.691 | 682.1392 |
| TCGA-DS-A1OD-01A-11R-A14Y-07 | -194.264 | 2416.67 | 2222.406 |
| TCGA-ZJ-AAX4-01A-11R-A42T-07 | -427.475 | 1692.61 | 1265.136 |
| TCGA-MY-A913-01A-11R-A37O-07 | -905.553 | 1110.884 | 205.331 |
| TCGA-UC-A7PF-01A-11R-A352-07 | -299.216 | 1927.419 | 1628.203 |
| TCGA-C5-A2LY-01A-31R-A18M-07 | -631.759 | 2190.967 | 1559.208 |
| TCGA-VS-A9UU-01A-11R-A42T-07 | -1607.77 | 122.4815 | -1485.29 |
| TCGA-C5-A8ZZ-01A-11R-A37O-07 | 256.8087 | -205.932 | 50.87691 |
| TCGA-EK-A3GJ-01A-21R-A213-07 | -1062.65 | 1577.802 | 515.1508 |
| TCGA-C5-A1MI-01A-11R-A14Y-07 | -851.232 | 710.3056 | -140.926 |
| TCGA-EA-A6QX-01A-12R-A33Z-07 | -841.906 | 1117.254 | 275.348 |
| TCGA-C5-A7X3-01A-11R-A352-07 | -302.351 | 1345.595 | 1043.244 |
| TCGA-EA-A3HT-01A-61R-A21T-07 | 133.8901 | 901.1655 | 1035.056 |
| TCGA-UC-A7PG-06A-11R-A42S-07 | -787.05 | 1517.117 | 730.067 |
| TCGA-VS-A9UB-01A-22R-A42T-07 | -865.069 | 2240.049 | 1374.98 |
| TCGA-VS-A8EK-01A-12R-A37O-07 | -1587.89 | 399.1038 | -1188.79 |
| TCGA-VS-A9UC-01A-11R-A42T-07 | -481.145 | 1092.2 | 611.0551 |
| TCGA-VS-A958-01A-11R-A42T-07 | -756.597 | 2265.094 | 1508.497 |
| TCGA-EK-A2R7-01A-11R-A18M-07 | -972.045 | 1064.753 | 92.70798 |
| TCGA-EA-A5ZD-01A-11R-A28H-07 | -444.213 | 1428.615 | 984.4021 |
| TCGA-C5-A1BQ-01C-11R-A213-07 | -879.64 | 1412.534 | 532.8944 |
| TCGA-JW-A5VI-01A-11R-A28H-07 | -402.622 | 973.4543 | 570.8326 |
| TCGA-ZJ-AAXF-01A-31R-A42T-07 | -220.755 | 1614.222 | 1393.466 |
| TCGA-EK-A2RK-01A-11R-A18M-07 | -1179.63 | 1207.26 | 27.63236 |
| TCGA-LP-A4AX-01A-12R-A24H-07 | -279.913 | 1975.908 | 1695.994 |
| TCGA-Q1-A5R3-01A-11R-A28H-07 | -1251.93 | 1201.974 | -49.9557 |
| TCGA-JX-A3Q0-01A-11R-A32Y-07 | -951.707 | 1411.686 | 459.9794 |
| TCGA-EX-A3L1-01A-11R-A32Y-07 | -496.547 | 1082.169 | 585.6211 |
| TCGA-EA-A3HS-01A-11R-A213-07 | -834.561 | 705.2609 | -129.301 |
| TCGA-C5-A2LT-01A-11R-A18M-07 | -288.692 | 1227.683 | 938.9911 |
| TCGA-ZX-AA5X-01A-11R-A42T-07 | 630.1026 | 1534.485 | 2164.588 |
| TCGA-EA-A439-01A-11R-A24H-07 | 36.02336 | 173.4195 | 209.4429 |
| TCGA-C5-A7CL-01A-11R-A32P-07 | -736.006 | 518.6427 | -217.363 |
| TCGA-C5-A1MF-01A-11R-A13Y-07 | -636.19 | 1299.452 | 663.2621 |
| TCGA-VS-A8EL-01A-11R-A37O-07 | -308.194 | 2037.162 | 1728.968 |
| TCGA-EK-A2GZ-01A-11R-A180-07 | -1404.58 | 579.7637 | -824.817 |
| TCGA-VS-A8QM-01A-11R-A37O-07 | 52.79915 | 2162.646 | 2215.445 |
| TCGA-HM-A3JJ-01A-21R-A21T-07 | 46.45428 | 1126.954 | 1173.408 |
| TCGA-XS-A8TJ-01A-11R-A36F-07 | -681.572 | 853.1302 | 171.5583 |

Table S2. The IRGPs with significant prognostic differences in univariate Cox proportional hazards regression analysis.

| IRGPs | HR | HR.95L | HR.95H | coxPvalue |
| --- | --- | --- | --- | --- |
| ABI3\|LPXN | 1.298247 | 0.961869 | 1.75226 | 0.08803 |
| ABI3\|SLAMF8 | 1.329047 | 0.988178 | 1.787499 | 0.059929 |
| AGAP2\|SIGLEC1 | 1.32028 | 0.965347 | 1.805713 | 0.082 |
| APOBEC3H\|FASLG | 1.297334 | 0.971257 | 1.732886 | 0.077985 |
| APOBEC3H\|TIGIT | 1.343993 | 0.995982 | 1.813605 | 0.053159 |
| BTLA\|GPR174 | 1.331468 | 0.970905 | 1.825933 | 0.075615 |
| C1QC\|SRGN | 0.703586 | 0.498019 | 0.994005 | 0.046144 |
| CD244\|GPR174 | 1.601478 | 1.135259 | 2.259161 | 0.007305 |
| CD28\|FASLG | 1.475711 | 1.04723 | 2.079508 | 0.026171 |
| CD37\|KLRB1 | 0.664966 | 0.479226 | 0.922696 | 0.014632 |
| CD3G\|LILRB4 | 1.406551 | 1.012435 | 1.954086 | 0.041988 |
| CD80\|RGS18 | 0.710201 | 0.532469 | 0.947256 | 0.019875 |
| CD84\|P2RY13 | 0.774517 | 0.58096 | 1.032561 | 0.081585 |
| CERKL\|RHOH | 0.695147 | 0.505226 | 0.956462 | 0.025525 |
| CLEC2D\|SIGLEC1 | 1.28703 | 0.968554 | 1.710227 | 0.081915 |
| CLEC4A\|LILRB4 | 1.308884 | 0.982898 | 1.742985 | 0.065486 |
| CMKLR1\|KLRB1 | 0.771839 | 0.567721 | 1.049346 | 0.098412 |
| CYTH4\|STAC3 | 0.75158 | 0.561535 | 1.005943 | 0.054842 |
| EVI2B\|SLAMF7 | 0.744246 | 0.554635 | 0.998678 | 0.048978 |
| FCRL3\|LY9 | 0.689625 | 0.515199 | 0.923103 | 0.012497 |
| FGR\|IL10RA | 1.427984 | 1.064371 | 1.915816 | 0.017501 |
| FOXP3\|RASAL3 | 0.687397 | 0.516233 | 0.915311 | 0.010298 |
| FUT7\|TFEC | 1.317401 | 0.990435 | 1.752305 | 0.058234 |
| GPR18\|IFNG | 1.304994 | 0.960663 | 1.772743 | 0.088532 |
| GPR34\|STAC3 | 1.427939 | 1.03564 | 1.968841 | 0.029732 |
| GYPC\|LST1 | 1.392671 | 0.987195 | 1.964691 | 0.05922 |
| IGSF6\|TNFSF13B | 1.302466 | 0.967437 | 1.753519 | 0.08155 |
| KLRB1\|PLA2G7 | 1.32604 | 0.969992 | 1.81278 | 0.076898 |
| LCP2\|SLAMF8 | 1.656778 | 1.185318 | 2.315759 | 0.003126 |
| LY9\|ZC3H12D | 1.337848 | 0.97285 | 1.839787 | 0.073353 |
| NLRC3\|SIGLEC1 | 1.445097 | 1.064468 | 1.961831 | 0.01825 |
| P2RY13\|SIGLEC1 | 1.32826 | 0.965314 | 1.827668 | 0.081302 |
| RGS18\|TBX21 | 1.327959 | 0.974286 | 1.810016 | 0.072638 |
| SAMD9L\|TMEM140 | 0.676018 | 0.477391 | 0.957286 | 0.027391 |
| SLAMF8\|ZNF683 | 0.737174 | 0.538394 | 1.009346 | 0.057179 |
